# Pyrophosphate Stimulates the Phosphate-Sodium Symporter of *Trypanosoma brucei* Acidocalcisomes and *Saccharomyces cerevisiae* Vacuoles

**DOI:** 10.1128/mSphere.00045-19

**Published:** 2019-04-03

**Authors:** Evgeniy Potapenko, Ciro D. Cordeiro, Guozhong Huang, Roberto Docampo

**Affiliations:** a Center for Tropical and Emerging Global Diseases, University of Georgia, Athens, Georgia, USA; b Department of Cellular Biology, University of Georgia, Athens, Georgia, USA; University of Texas Southwestern

**Keywords:** SPX domain, *Saccharomyces cerevisiae*, *Trypanosoma brucei*, *Xenopus laevis*, acidocalcisome, phosphate-sodium symporter, pyrophosphate

## Abstract

Inorganic pyrophosphate (PP_i_) is a by-product of biosynthetic reactions and has bioenergetic and regulatory roles in a variety of cells. Here we show that PP_i_ and other pyrophosphate-containing compounds, including polyphosphate (polyP), can stimulate sodium-dependent depolarization of the membrane potential and P_i_ conductance in *Xenopus* oocytes expressing a Saccharomyces cerevisiae or Trypanosoma brucei Na^+^/P_i_ symporter. PP_i_ is not taken up by *Xenopus* oocytes, and deletion of the TbPho91 SPX domain abolished its depolarizing effect. PP_i_ generated outward currents in Na^+^/P_i_-loaded giant vacuoles prepared from wild-type or *pho91*Δ yeast strains expressing *TbPHO91* but not from the *pho91Δ* strains. Our results suggest that PP_i_, at physiological concentrations, can function as a signaling molecule releasing P_i_ from S. cerevisiae vacuoles and T. brucei acidocalcisomes.

**IMPORTANCE** Acidocalcisomes, first described in trypanosomes and known to be present in a variety of cells, have similarities with S. cerevisiae vacuoles in their structure and composition. Both organelles share a Na^+^/P_i_ symporter involved in P_i_ release to the cytosol, where it is needed for biosynthetic reactions. Here we show that PP_i_, at physiological cytosolic concentrations, stimulates the symporter expressed in either *Xenopus* oocytes or yeast vacuoles via its SPX domain, revealing a signaling role of this molecule.

## Abstract (precis)

Acidocalcisomes, first described in trypanosomes and known to be present in a variety of cells, have similarities with S. cerevisiae vacuoles in their structure and composition. Both organelles share a Na^+^/P_i_ symporter involved in P_i_ release to the cytosol, where it is needed for biosynthetic reactions. Here we show that PP_i_, at physiological cytosolic concentrations, stimulates the symporter expressed in either *Xenopus* oocytes or yeast vacuoles via its SPX domain, revealing a signaling role of this molecule.

## INTRODUCTION

Inorganic pyrophosphate (PP_i_) is a side product of more than 200 biosynthetic reactions, like the synthesis of isoprenoids, nucleic acids, proteins, and coenzymes and the activation of fatty acids (1). Hydrolysis of PP_i_ by pyrophosphatases (PPases) has been recognized to make these biosynthetic reactions thermodynamically favorable (2). PP_i_ also has bioenergetic functions and regulatory roles for several enzymes and processes (3), although a signaling role has not been considered. PP_i_ can be generated by glycolysis, oxidative phosphorylation, and photophosphorylation and can replace ATP in a number of reactions (4). The cytosolic concentration of PP_i_ is regulated in eukaryotic cells by soluble PPases (5).

An unusual characteristic of Trypanosoma brucei, the etiologic agent of sleeping sickness or African trypanosomiasis, and of other trypanosomatids is that they possess higher cellular levels of PP_i_ than of ATP (6). Most PP_i_, as well as polyphosphate (polyP), is stored in acidic organelles named acidocalcisomes (7). Acidocalcisomes from T. brucei are electron dense and possess large amounts of cations bound to polyP, with several pumps in their membranes, like the vacuolar proton pyrophophatase (V-H^+^-PPase), which contributes to their acidification (8). When fixed Trypanosoma cruzi (9) or Trypanosoma evansi (10) cells are treated with PPase, the electron-dense matrix of acidocalcisomes is removed, indicating that PP_i_ is a component of this organelle’s structure. Besides the acidocalcisomal V-H^+^-PPase, other enzymes of T. brucei, such as a soluble pyrophosphatase (11–13), and the glycosomal pyruvate-phosphate dikinase (14) can use PP_i_.

Recent work has shown that a phosphate-sodium symporter from both T. brucei acidocalcisomes (TbPho91) and Saccharomyces cerevisiae vacuoles (Pho91p) is stimulated to release P_i_ and Na^+^ to the cytosol by the binding of inositol hexakisphosphate (IP_6_) or diphosphoinositol pentakisphosphate (5-PP-IP_5_ or 5-IP_7_) to their SPX domain (15). PP_i_ is formed by biosynthetic reactions, like the synthesis of deoxynucleotide triphosphates (dNTPs) that are needed for yeast DNA duplication (16) or for the biosynthesis of phospholipids and nucleotides needed for cell duplication (17). These reactions require an abundant source of P_i_. We therefore considered a potential signaling role of PP_i_ in the export of vacuolar P_i_. Heterologously expressed *TbPHO91*, with or without its SPX domain, in *Xenopus* oocytes was tested by the two-electrode voltage clamp method to measure transmembrane currents in the presence of PP_i_ and polyphosphates. We also prepared giant vacuoles of yeast expressing either wild-type or T. brucei Na^+^/P_i_ symporters and patch-clamped them. We report that PP_i_ stimulates TbPho91 and Pho91p, leading to P_i_ and Na^+^ release to the cytosolic side of the vacuoles, and that the presence of an SPX domain in TbPho91 is important for this stimulation to occur.

## RESULTS

### Modulation of the Na^+^/P_i_ conductance of TbPho91 by pyrophosphate and polyphosphates.

**FIG 1:**
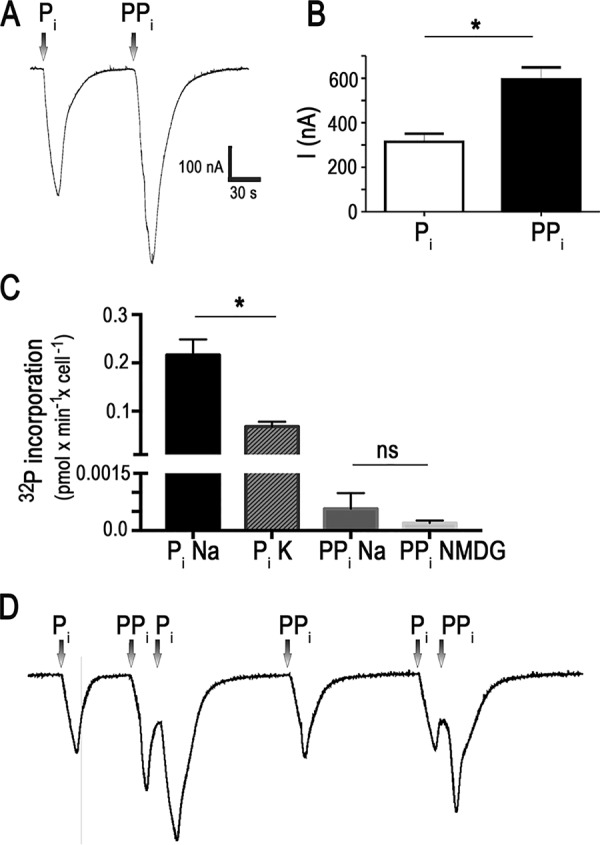
Effect of PP_i_ on P_i_-elicited currents in oocytes expressing TbPho91 and P_i_ and PP_i_ uptake by the same oocytes. (A) Representative currents recorded after the addition of 10 mM Na^+^/P_i_ or 10 mM Na^+^/PP_i_ to oocytes expressing TbPho91. (B) Quantification of results from several experiments as described for panel A. (C) ^32^P incorporation of Na^+^/^32^P_i_, K^+^/^32^P_i_, Na^+^/^32^PP_i_, or NMDG/^32^PP_i_ into oocytes expressing TbPho91. (D) Representative currents after sequential addition of 10 mM Na^+^/P_i_ or 10 mM Na^+^/PP_i_ to oocytes expressing TbPho91. Values in panels B and C are means ± SEM; *n* = 6 (B) and *n* = 3 (C). *, P < 0.05 (Student's *t* test); ns, not significant.

TbPho91 localizes to acidocalcisomes (18), and these organelles are rich in PP_i_ (6). Therefore, we examined whether this compound induced net inward currents when applied to *Xenopus* oocytes expressing the symporter. Figure 1A shows the inward current generated at holding potential (*V_h_* = −60 mV) by the addition of equimolar concentrations of P_i_ or PP_i_. The current amplitude induced by PP_i_ was a few hundred nanoamperes and was larger than that induced by P_i_ (Fig. 1A and B). One possible reason for the induction of these inward currents is the cotransport of Na^+^ and PP_i_, through TbPho91. However, while there is Na^+^-dependent uptake of ^32^P_i_, there is no significant Na^+^-dependent ^32^PP_i_ uptake into oocytes expressing TbPho91 (Fig. 1C). The results suggest that while PP_i_ is not transported, Na^+^ transport, which generates an inward current, is stimulated by PP_i_. Interestingly, when PP_i_ was added before P_i_, P_i_ induced larger current amplitudes than when added alone, indicating that PP_i_ has a modulating effect on Na^+^ transport through TbPho91 (Fig. 1D).

### PolyPs of different lengths induce inward currents in a pH- and calcium-dependent manner in oocytes expressing *TbPho91 and PHO91*.

**FIG 2:**
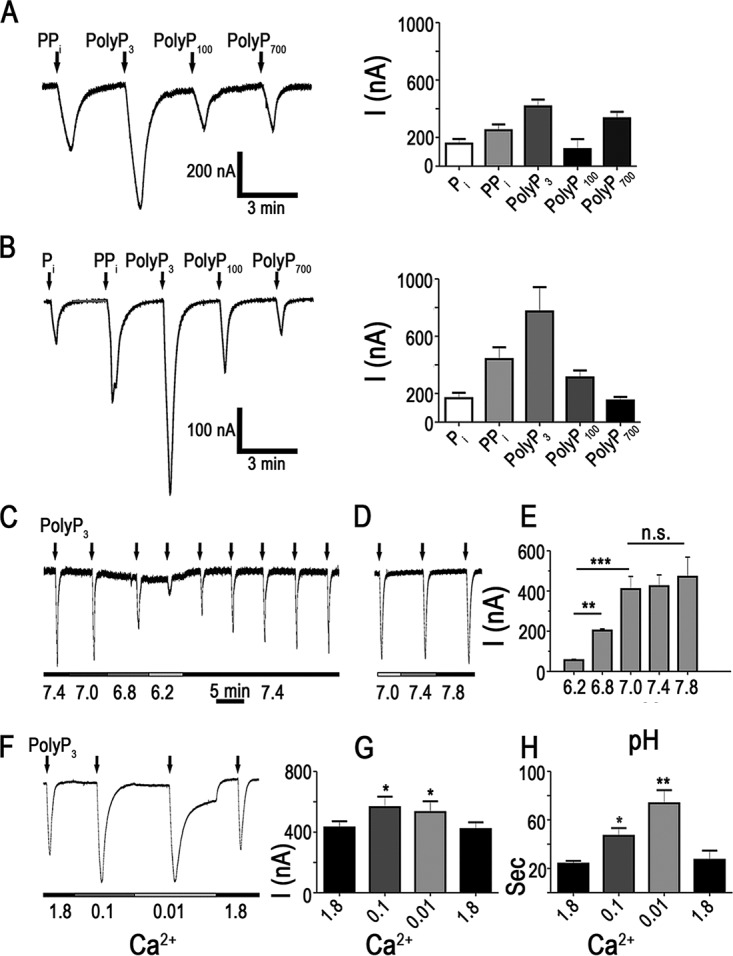
Currents elicited by PP_i_ and polyPs in oocytes expressing TbPho91 and Pho91p. (A) Representative currents recorded after the addition of 10 mM Na^+^/PP_i_, Na^+^/polyP_3_, Na^+^/polyP_100_, and Na^+^/polyP_700_ to oocytes expressing TbPho91. The right panel shows the quantification of currents elicited from four experiments. (B) Representative currents recorded after the addition of 10 mM Na^+^/PP_i_, Na^+^/polyP_3_, Na^+^/polyP_100_, and Na^+^/polyP_700_ to oocytes expressing Pho91p. The right panel shows the quantification of currents elicited from four experiments. (C to E) Currents recorded in response to the addition of 10 mM Na^+^/polyP_3_ at different pH levels (C and D) and quantification of the results of three experiments (E). (F to H) Currents recorded in response to the addition of 10 mM Na^+^/polyP_3_ at different Ca^2+^ concentrations (F) and quantification of the current intensity (G) or current duration (H) of several experiments. Values in panels E, G, and H are means ± SEM; *n* = 4. *, P < 0.05; **, P < 0.01; ***, P < 0.001; n.s., not significant (Student's *t* test). Concentrations of Na^+^/polyP_100_ and Na^+^/polyP_700_ are expressed in phosphate units.

When polyPs of different lengths were used, similar inductions of inward currents were observed. PolyP_3_ (tripolyphosphate or TPP) induced currents of larger amplitude than polyP_100_ or polyP_700_ (Fig. 2A), and similar results were observed when S. cerevisiae Na^+^/P_i_ cotransporter (*PHO91*) was expressed in oocytes (Fig. 2B). However, when we used the same concentration of PP_i_ and polyP_3_ in phosphate units as with the longer polyPs, the amplitude changes were not significantly different (data not shown). Peak amplitudes of inward currents in oocytes expressing *TbPho91* (in nanoamperes) were as follows: 250.1 ± 40.4 (*n* = 4) for PP_i_, 416.3 ± 47.4 (*n* = 4) for polyP_3_, 119 ± 69.6 (*n* = 5) for polyP_100_, and 333.5 ± 45.6 (*n* = 4) for polyP_700_ (Fig. 2A, right panel). In oocytes expressing yeast *PHO91*, the amplitudes of inward currents (in nanoamperes) were as follows: 447.8 ± 84.1 (*n* = 5) for PP_i_, 771.6 ± 168.4 (*n* = 5) for polyP_3_, 312.4 ± 50.8 (*n* = 5) for polyP_100_, and 142.8 ± 23.5 (*n* = 5) for polyP_700_ (Fig. 2B, right panel). The control amplitudes of Na^+^/P_i_ currents in *TbPho91*- and *PHO91*-expressing oocytes were 157.1 ± 32.8 nA and 146.8 ± 41.6 nA (*n* = 4), respectively (Fig. 2A and B, right panels).

Similar to P_i_-induced currents (15), polyP-induced currents also depended on extracellular pH (Fig. 2C). Acidification of the extracellular medium inhibited TbPho91 currents induced by the application of 10 mM polyP_3_. The amplitude of the Na^+^/polyP transient was significantly lower at pH 6.8 (203.5 ± 12 nA, *P* < 0.0001, *n* = 8) and pH 6.2 (56 ± 5.7 nA, *P* < 0.0001, *n* = 8) than at pH 7.0 (424.1 ± 65 nA). This effect was reversible in the course of tens of minutes in medium at neutral pH. However, a shift to more alkaline pH values (up to pH 7.8) did not produce significant changes in current (Fig. 2D). Figure 2E shows means ± standard errors of the means (SEM) of the results of three experiments.

Decreasing the extracellular calcium concentration ([Ca^2+^]_out_) from 1.8 mM to 100 and 10 µM induced an increase in polyP_3_ current from 426.4 ± 36.6 nA to 564.8 ± 41 nA (*P* < 0.05, *n* = 8) and 534.8 ± 69.6 nA (*P* < 0.05, *n* = 4), respectively (Fig. 2F and G). In addition, the kinetics of the polyP_3_ current transient was also changed, showing a slow decay in restoration to the basal level, especially at 10 µM [Ca^2+^]_out_ (Fig. 2F). Thus, the half-width of the current transient increased (in seconds) from 23.9 ± 2.3 at 1.8 [Ca^2+^]_out_ to 46.8 ± 6.4 (*P* < 0.05, *n* = 4) and 73.7 ± 10.8 (*P* < 0.01, *n* = 4) at 100 µM and 10 µM [Ca^2+^]_out_, respectively (Fig. 2H). An increase of [Ca^2+^]_out_ above 3.0 mM led to oocyte death within minutes.

Taken together, the results suggest that PP_i_ and polyPs might be modulating the opening of the Na^+^/P_i_ cotransporter and facilitating Na^+^ transport and generation of the currents in a pH- and calcium-dependent manner.

### Modulation of the Na^+^/P_i_ conductance of TbPho91 by pyrophosphate is dependent on the SPX domain.

It has been recognized that the SPX domains present in the N termini of vacuolar transporter chaperones, signaling proteins, and phosphate transporters can function as polyphosphate sensor domains (19). They bind to phosphate-containing ligands like PP_i_, polyP_3_, and IP_6_ at micromolar levels and to 5-IP_7_ at nanomolar concentrations. Similarly, we found that IP_6_ and 5-IP_7_ stimulate the yeast and T. brucei Na^+^/P_i_ symporter through its SPX domain (15). We therefore investigated whether this was also the case with PP_i_.

**FIG 3:**
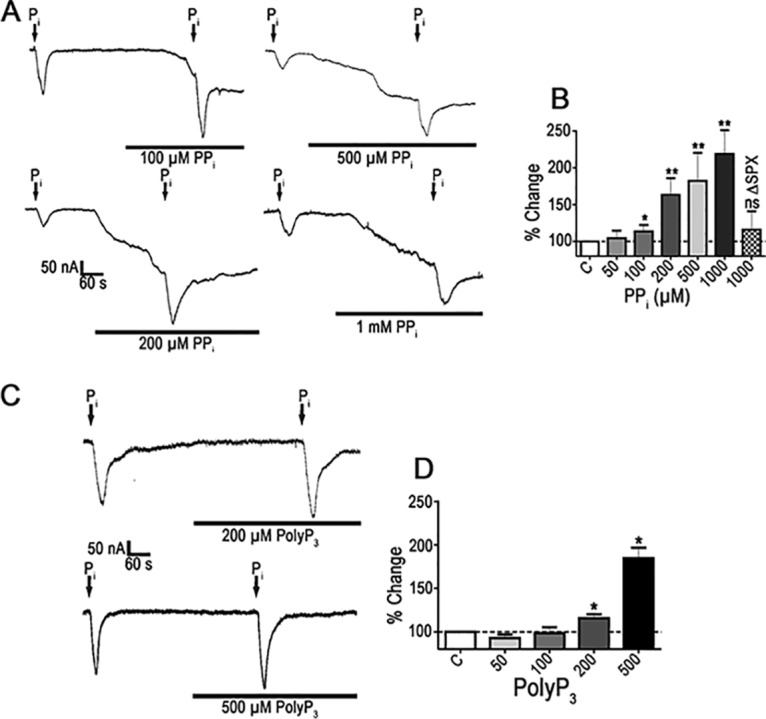
Effect of PP_i_ on P_i_-elicited currents in oocytes expressing TbPho91. (A) Representative currents recorded when the addition of 10 mM Na^+^/P_i_ was done in the absence or presence of the indicated concentrations of PP_i_. (B) Quantification of the results of several experiments as described for panel A. C, control. (C) Representative currents recorded when the addition of 10 mM Na^+^/P_i_ was done in the absence or presence of the indicated concentrations of polyP_3_. (D) Quantification of the results of several experiments as described for panel C. C, control. Values in panels B and D are means ± SEM; *n* = 4. *, *P* < 0.05; **, P < 0.01 (Student's *t* test).

In addition to the ability of PP_i_ to directly activate TbPho91, it can also modulate the Na^+^/P_i_ current. When oocytes were preincubated for 5 to 6 min with PP_i_, in the micromolar range (Fig. 3A), there was an induction of slow inward currents, followed by amplification of the Na^+^/P_i_-transmembrane current evoked by 10 mM P_i_. The thresholds for statistically significant amplification of the Na^+^/P_i_ current were 100 μM for PP_i_ (13.5% ± 0.87% higher than the reference value, *P* < 0.05, *n* = 5) (Fig. 3B) and 200 μM for polyP_3_ (+15.7%, *P* < 0.05, *n* = 4) (Fig. 3C and D).

To examine the role of the SPX domain of TbPho91 in this stimulation by PP_i_, we expressed the protein with a deletion of this domain (TbPho91-ΔSPX) (15) and measured its response to PP_i_. When *TbPho91-*Δ*SPX*-expressing oocytes were tested, no amplification of the currents induced by 10 mM P_i_ occurred by the addition of PP_i_ (Fig. 3B), which confirms previous findings on the role of the SPX domain in regulating Phop91p conductance.

### Stimulation of Na^+^/P_i_ release by PP_i_ from yeast vacuoles.

We applied the spheroplast incubation method to prepare giant cells of S. cerevisiae by using 2-deoxyglucose to inhibit cell wall synthesis (20). The giant cells were treated by moderate hyposmotic shock to disrupt the plasma membrane and release the enlarged vacuoles. A patch pipette was then attached to the vacuolar membrane, and after formation of a gigaseal, the patch membrane was ruptured by high-voltage pulses. The lumen of the vacuole was connected to the pipette (whole-vacuole configuration) and was loaded with a solution containing Na^+^ and P_i_, to record transmembrane currents. We used *pho91Δ* cells to express *TbPHO91*.

**FIG 4:**
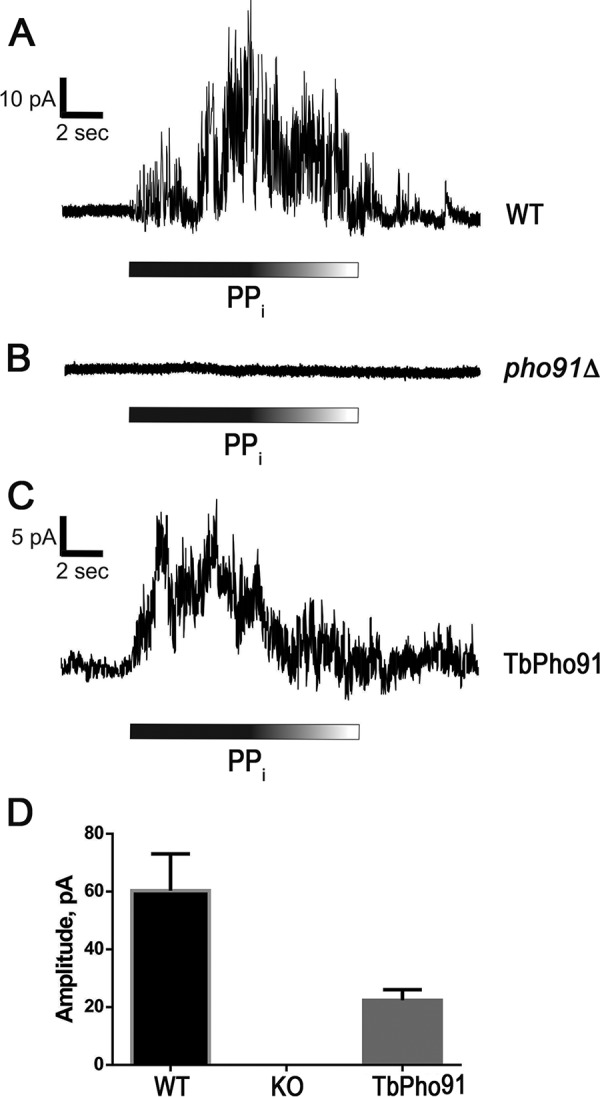
PP_i_ induces activation of Na^+^/Pi currents in Pho91- and TbPho91-expressing yeast vacuoles. (A) Activation of Na^+^/Pi outward currents in vacuoles from wild-type yeast after the addition of 10 mM PP_i_. (B) *pho91Δ* vacuoles do not produce currents after application of PP_i_. (C) Complementation of *pho91Δ* with *TbPHO91* restores vacuole response to PP_i_. Data are representative of two to four independent experiments and are quantified in panel D. We used at least four successful current recordings for each experiment. About 80% of the vacuoles showed clear responses. WT, wild type; KO, knockout.

Patch-clamp recordings of the vacuoles were performed at a *V_h_* of +60 mV. The bath solution had 10 mM HEPES, pH 7.1, containing 100 mM NaCl, 200 mM sorbitol, and 1 mM MgCl_2_, while the pipette solution contained a similar solution plus 10 mM NaH_2_PO_4_-Na_2_HPO_4_ in order to detect outward currents generated by displacement of Na^+^/P_i_ to the bath solution (“cytosol”). After 10 mM PP_i_ was added to the bath solution (Fig. 4A), we registered outward currents of 60.3 ± 12.7 pA (*n* = 3) in vacuoles from wild-type cells. When vacuoles from *pho91Δ* cells were used, no significant currents were detected after PP_i_ application (Fig. 4B).

We then expressed *TbPHO91* in giant vacuoles of *pho91Δ* cells. Application of 10 mM PP_i_ induced outward currents of 22.3 ± 3.7 pA (*n* = 3) (Fig. 4C and D). Our results demonstrate that PP_i_ triggers the release of Na^+^/P_i_ by the Pho91 symporters.

## DISCUSSION

We report here that functional expression in Xenopus laevis oocytes of T. brucei or S. cerevisiae Na^+^/P_i_ symporter Pho91, followed by two-electrode voltage clamp recordings, showed that the application of PP_i_ or polyP resulted in the depolarization of the oocyte membrane potential and an increase in the P_i_ conductance. The stimulation induced by PP_i_ was abolished when the SPX domain of the symporter was deleted. Application of PP_i_ to yeast giant vacuoles expressing TbPho91 or Pho91p but not to vacuoles of *pho91Δ* cells induced outward currents, suggesting a role of PP_i_ in Na^+^/P_i_ release.

Pyrophosphate does not penetrate *Xenopus* oocytes, but it stimulates the Pho91 transporters that are expressed in them. If this happens through the SPX domain, the domain would have to be oriented toward the exterior of the oocyte. Plasma membrane orientation is essentially demonstrated by positive functionality. The best evidence that the topology of Pho91 and TbPho91 in *Xenopus* oocytes is inverted is that Na^+^ and P_i_ are transported into the oocytes, as demonstrated by electrophysiological recordings and ^32^P_i_ uptake experiments. This does not occur in the giant vacuoles, where we detected P_i_ release to the cytosolic side of the vacuole. The currents detected are due to the electrogenic nature of the transporter (Na^+^ is the charge carrier, and P_i_ without Na^+^ does not elicit currents [15]). The transfer of Na^+^ to the cytosol is favored by the higher Na^+^ concentration in the extracellular medium. In contrast, acidocalcisomes and yeast vacuoles have more Na^+^ than the cytosol and Na^+^ efflux is favored. This inversion of the membrane topology in the plasma membrane of *Xenopus* oocytes indicates that the amino-terminal region containing the SPX domain is also inverted and oriented toward the outside, as demonstrated by the experiments with expression of truncated TbPho91. This is also in agreement with structural data available for other P_i_ transporters (21) that showed that there is no reorientation of the carrier alternatively exposing the substrate binding sites to one or the other side of the membrane, as previously postulated (22, 23), but movement of ions within the transmembrane field. It is known that lipid composition can affect topology of a membrane protein, or orientation of its α-helices in a membrane, which underlies membrane protein function. Inversion of the membrane topology of vacuolar transporters expressed in the plasma membrane of *Xenopus* oocytes is not infrequent (24).

Our results concerning the role of the SPX domain in the yeast Pho91p is at variance with its role in the plasma membrane low-affinity P_i_ transporters Pho87p and Pho90p (25). When the SPX domain was removed to generate a truncated form of Pho90p, there was increased accumulation of phosphate, which was proposed as evidence that SPX is a regulatory domain that inhibits phosphate transport under normal conditions (25). However, the SPX removal experiments did not provide mechanistic evidence on how SPX regulates the transporters. Electrophysiological characterization of Pho90p and Pho87p could reveal whether polyphosphate-containing molecules (or the Slp2 protein), acting on the SPX domain, regulate phosphate uptake by these low-affinity transporters.

It was shown before that 1 mM PP_i_ “primes” (26) or stimulates the catalytic domain of the polyP polymerase vacuolar transporter chaperone 4 (VTC4) of S. cerevisiae but inhibits the catalytic domain of T. brucei VTC4 (27). PP_i_ and polyP_3_ also bind to the SPX domain of S. cerevisiae VTC2, as determined by isothermal titration calorimetry, with dissociation constants (*K_d_*) of 154 ± 62 and 11.1 ± 1.7 µM, respectively, but PP_i_ does not significantly stimulate VTC-catalyzed polyP synthesis by isolated yeast vacuoles at millimolar concentrations (26). We found that the threshold for PP_i_ for statistically significant amplification of the Na^+^/P_i_ current in *Xenopus* oocytes expressing *TbPho91* was 100 μM, which is within the physiological levels of cytosolic PP_i_ in several cell types. For example, the PP_i_ concentration in the cytosol of plant cells is about 0.2 to 0.3 mM (28), while in exponentially growing Escherichia coli K-12 cells, the intracellular PP_i_ concentration is about 0.5 mM, even after varying the amount of pyrophosphatase from 15 to 2,600% of the control amount (29). In addition, the PP_i_ content in E. coli can be increased up to 2.5 mM when the growth of cells is limited by inhibition of the synthesis of nucleotides (30). The concentration of PP_i_ in different species has been reviewed extensively, and, for example, it has been estimated to be at about 100 to 200 µM in rat liver (3).

Although polyP_3_ and other polyPs are able to induce inward currents in *Xenopus* oocytes expressing *TbPho91*, their physiological relevance is relative, as most of these compounds are compartmentalized in the acidocalcisomes (6, 7), nucleolus, and glycosomes (31). In this regard, it has been demonstrated the polyP is toxic when in the yeast cytosol (32). We do not think that polyPs could have a physiological role, and we attribute their stimulatory effect to their chemical similarities to PP_i_.

**FIG 5:**
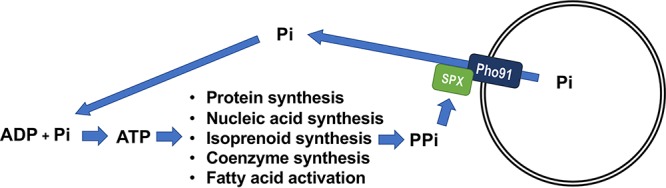
Schematic representation of PP_i_ signaling function. Large amounts of P_i_ are needed for biosynthetic pathways, which generate PP_i_ as a by-product. PP_i_ stimulates the vacuolar Pho91 Na^+^/P_i_ symporters through their SPX domains, increasing the release of P_i_ needed for ATP biosynthesis.

In conclusion, our work revealed that PP_i_ stimulates the Na^+^/P_i_ symporter of T. brucei acidocalcisomes, and that of its yeast ortholog localized in the vacuole, through its SPX domain. This stimulation results in the release of P_i_ and Na^+^ to the cytosolic side of the vacuoles. Our hypothesis is that as result of enhanced PP_i_ production by anabolic reactions, the increase in PP_i_ would stimulate the P_i_ release needed for these anabolic reactions (Fig. 5). The results reveal an unrecognized role of PP_i_ in cell signaling.

## MATERIALS AND METHODS

### Chemicals and reagents.

Integrated DNA Technologies (Coralville, IA) provided the primers used. All other reagents of analytical grade were purchased from Sigma-Aldrich (St. Louis, MO).

### Cell cultures.

T. brucei (Lister 427 strain procyclic forms [PCF]) were grown at 28°C in SDM-79 medium (33) with 10% heat-inactivated fetal bovine serum and hemin (7.5 µg/ml).

### Yeast strains.

We used S. cerevisiae strain BY4741 (*MATa his3Δ1 leu2Δ0 met15Δ0 ura3Δ0*). Generation of *pho91Δ* was as described previously (15).

### Preparation and isolation of giant yeast vacuoles.

Preparation of giant yeast vacuoles from the wild type and *pho91*Δ mutants was done as described before (20), with minor modifications (15). The vacuoles were attached to a poly-l-lysine-coated chamber for patch-clamp recording. The micropipette solution contained 10 mM HEPES, pH 7.1, 100 mM NaCl, 200 mM sorbitol, 1 mM MgCl_2_, 5 mM NaH_2_PO_4_, and 5 mM Na_2_HPO_4_. The bath solution was similar, but without NaH_2_PO_4_, and Na_2_HPO_4_.

### Preparation and maintenance of oocytes.

Xenopus laevis oocytes were obtained from Xenoocyte (Dexter, MI). Oocytes collected at stage IV or V were manually defolliculated and devitellinized with collagenase (1 mg/ml) for 1 h at room temperature and then maintained in filtered modified Barth’s solution containing 88 mM NaCl, 1 mM KCl, 0.82 mM MgSO_4_, 0.41 mM CaCl_2_, 2.4 mM NaHCO_3_, 0.33 mM Ca(NO_3_)_2_, and 10 mM HEPES, plus 50 µg/ml gentamicin, pH 7.4, at a density of less than 100 per 60-mm plastic petri dish. Barth’s solution was replaced daily.

### cRNA production, oocyte injection, and electrophysiology.

PrimeSTAR HS DNA polymerase (Clontech) was used to amplify by PCR full-length *TbPHO91* (Tb427tmp.01.2950), truncated *TbPHO91* (*TbPHO91-ΔSPX*; obtained by removal of the 606-nucleotide sequence encoding the N-terminal putative SPX domain), and *PHO91* (GenBank accession number NM_001183190) open reading frames (15) from T. brucei or S. cerevisiae genomic DNA, using the corresponding gene-specific primers indicated in Table 1. The PCR products were purified as described previously (15), and the nucleotide sequences were confirmed by sequencing. cRNAs were obtained by *in vitro* transcription using the purified PCR products as the templates with an mMESSAGE mMACHINE kit (Ambion Life Technologies, Thermo Fisher Scientific, Inc, Waltham, MA), in accordance with the manufacturer’s protocol, and verified as described previously (15). cRNA injection was done exactly as described before (15). Equal amounts of cRNA from control and mutant transporters were injected into the *Xenopus* oocytes. For electrophysiology, the standard two-electrode voltage-clamp technique was used, as described previously (15). At least four oocytes from two different frogs were used in each experiment. All recordings were obtained at room temperature. Oocytes were bathed in ND96 buffer bath solution containing 96 mM NaCl, 2 mM KCl, 5 mM MgSO_4_, 1 mM CaCl_2_, and 2.5 mM HEPES, pH 7.5, with a continuous perfusion speed of ∼2 ml/min. Low-calcium solutions were prepared by adding Ca^2+^ and EGTA at proportions calculated with MaxChelator software (Stanford University, CA). The required pH of ND96 was adjusted either with NaOH or HCl. The effect of PP_i_ and polyphosphates was studied by their addition to ND96 with subsequent pH readjustments. To prepare the phosphate solution, 300 mM stock solutions of mono- and dibasic sodium phosphates were mixed until pH 7.4 was obtained.

**TABLE 1:** Primers used in this study

Primer sequence^*a*^	Use^*b*^
AGGAAAAATGCCGCTCAAAATCT	Knockout of yeast *PHO91*
CAATACAAATGGGCATTGACCAGA	Knockout of yeast *PHO91*
TTGGGTACCGGGCCCCCCCTCGAGGTGGGCCTATCCGCCTTAAT	Amplification of *PHO91* for cloning in pRS413
GGATCCCCCGGGCTGCAGGAATTCAATCATAAGTGGTGCGGCCA	Amplification of *PHO91* for cloning in pRS413
GACACGGTAACTTGCAGACTGACATGAAGTTCGGAAAGCG	Amplification of *TbPHO91* for fusing with *PHO91* UTRs and cloning in pRS413
TTTCATTCTCTCTATGGATAATCCTACGGTTTGCCTTCAAA	Amplification of *TbPHO91* for fusing with *PHO91* UTRs and cloning in pRS413
TTGGGTACCGGGCCCCCCCTCGAGGTGGGCCTATCCGCCTTAAT	Amplification of *PHO91* 5′ UTR for fusing with *TbPHO91*
GTCAGTCTGCAAGTTACCGTGTCACCTTCACAGTTTTCTTTTTATTTG	Amplification of *PHO91* 5′ UTR for fusing with *TbPHO91*
GATTATCCATAGAGAGAATGAAAGGTTACTAATATAGTATGTATACGTGC	Amplification of *PHO91* 3′ UTR for fusing with *TbPHO91*
GGATCCCCCGGGCTGCAGGAATTCAATCATAAGTGGTGCGGCCA	Amplification of *PHO91* 3′ UTR for fusing with *TbPHO91*
CCCGCGAAATTAATACGACTCACTATAGGGAGA**CCACC***ATGAAGTTCGGAAAGCGGC*	*TbPHO91T7F* (for *Xenopus* expression)
CCCGCGAAATTAATACGACTCACTATAGGGAGA**CCACC***ATGGAAGCAGAGATTAGCCG*	*TbPHO91TFN* (for *Xenopus* expression)
TTTTTTTTTTTTTTTTTTTTTTTTTTTTTT*CTACGGTTTGCCTTCAAACAC*	*TbPHO91T30R* (for *Xenopus* expression)
CCCGCGAAATTAATACGACTCACTATAGGGAGA**CCACC***ATGAAGTTCTCGCATTCCT*	*PHO91T7F* (for *Xenopus* expression)
TTTTTTTTTTTTTTTTTTTTTTTTTTTTTT*CTAAAATCCCATTACTTTCAATATGCC*	*PHO91T30R* (for *Xenopus* expression)

a For the last five primers, T_7_ promoter or polyT_30_ sequences are underlined. Kozak consensus sequences for increasing efficiency of translation initiation are in bold. Gene-specific sequences are italicized. Additional nucleotides upstream of the T_7_ promoter or the Kozak consensus sequence are incorporated into the primers for desirable *in vitro* transcription/translation in Xenopus laevis oocytes.

b UTR, untanslated region.

Yeast giant vacuole experiments were done exactly as described previously (15). All recordings were performed at a *V_h_* of +60 mV. An Axopatch 200b amplifier was used for current registration, and data were filtered at 1,000 Hz, digitized with Digidata 1550A (Axon Instruments, USA), and analyzed offline using PClamp 10 software.

### ^32^P and ^32^PP uptake assays.

Xenopus laevis oocytes were injected with cRNA as described above and used after 3 days. Oocytes were incubated in standard ND96 solution or a modified ND96 solution with sodium replaced by an equimolar concentration of potassium or NMDG (ND96–Na). The healthiest looking oocytes were transferred to Eppendorf tubes (6 per tube) and incubated with 200 µl of ND96 or ND96–Na solutions containing 300,000 cpm of inorganic ^32^P (60 Ci/mmol) or ^32^P-labeled pyrophosphate (60 Ci/mmol) (Perkin Elmer). Oocytes were then incubated for 30 min at room temperature and washed five times with 1 ml of ND96 or ND96–Na. Prolonged incubation of oocytes under these conditions decreased the oocyte quality, probably due to strong and long-lasting depolarization of the cellular membrane. Oocytes were then lysed with 10% sodium dodecyl sulfate (SDS), and the total lysate was added to the scintillation cocktail (MP Biomedicals). ^32^P radiation was measured using an LS 6500 multipurpose scintillation counter (Beckman Coulter). Each of three experiments was done using triplicate measurements.

### Statistical analysis.

All values are expressed as means ± SEM, unless indicated otherwise. Significant differences between treatments were compared using unpaired Student’s *t* tests. Differences were considered statistically significant at a *P* of <0.05, and *n* refers to the number of independent biological experiments performed. All statistical analyses were conducted using GraphPad Prism 6 (GraphPad Software, San Diego, CA).
